# Evaluation of Web-Based Health Information From the Perspective of Women With Eating Disorders: Thematic Analysis

**DOI:** 10.2196/31148

**Published:** 2022-06-13

**Authors:** Hana Drtilova, Hana Machackova, Martina Smahelova

**Affiliations:** 1 Interdisciplinary Research Team on Internet and Society Faculty of Social Studies Masaryk University Brno Czech Republic

**Keywords:** eating disorders, web-based health information, Czech women

## Abstract

**Background:**

Users with experience of eating disorders use the internet as a source of information, whether for prorecovery activities (such as web-based treatment, looking for information, support, and sharing) or activities that promote eating disorder behavior as a desirable lifestyle choice (such as pro–eating disorder communities and reading and creating pro–eating disorder posts). Their assessment of web-based eating disorder–related information is crucial for understanding the context of the illness and for health professionals and their web-based interventions.

**Objective:**

This study aimed to understand the criteria young women with the experience of eating disorders use in evaluating eating disorder–related web-based information and what eating disorder–related characteristics of these women are involved in their evaluation.

**Methods:**

We analyzed 30 semistructured individual interviews with Czech women aged 16 to 28 years with past or present eating disorder experience using a qualitative approach. Thematic analysis was adopted as an analytical tool.

**Results:**

The specifics of eating disorder phases (the *disorder stage* and the *treatment process*) emerged as important aspects in the process of information assessment. Other specific characteristics of respondents (eg, motivation, abilities, and resources) addressed how the respondents arrived at certain web-based information and how they evaluated it. In addition, the respondents described some content cues as features of information (eg, novelty and social information pooling). Another finding is that other users’ attitudes, experiences, activities, and personal features are involved in the information evaluation of these users and the information presented by them. Finally, the respondents evaluated the websites’ visual look and graphic components.

**Conclusions:**

This study shows that web-based information evaluation reported by women with experience of eating disorders is a complex process. The assessment is influenced by current personal characteristics related to the illness (mainly the motivation for maintaining or curing the eating disorder) using cues associated with information content, other users, and website look. The study findings have important implications for health professionals, who should ask their clients questions about web-based communities and their needs to understand what information and sources they choose.

## Introduction

### Background

During the past 2 decades, the internet has been integrated into our lives as an everyday tool that opens the gate to an unlimited amount of web-based information. In this content-rich environment with almost no quality control [1], the burden of information assessment shifts toward the information seeker. The internet provides user-generated content where nonspecialists offer health tips or information based on personal experiences [2], which can be highly relevant, especially in health-related web-based searches. Wilson et al [3] revealed that among people with eating disorders (EDs) experience, 75% used the internet as a source of information, 40.8% reported visiting prorecovery sites, and 35.5% reported visiting sites that promote ED behavior. Health providers often use the internet as a tool for prevention and as an environment for ED treatment programs [4]. Consequently, for people with ED experience, web-based information can be potentially beneficial and helpful (eg, web-based treatment [5]) but also dangerous and harmful (eg, anorexia-related misinformation on YouTube [6]). For these reasons, this study aimed to deepen the knowledge of users’ web-based activities with ED experience by examining their evaluation of web-based information relevant to ED topics. Specifically, the aim was to investigate the individual characteristics that shape this process and the cues used for information assessment.

### EDs and Internet Use

EDs are part of the spectrum of pathological eating patterns and are perceived as either a medical illness with psychiatric features or a psychiatric illness with medical indications [7]. Regardless of the subtypes (the most known are anorexia nervosa [AN], bulimia nervosa [BN], and binge ED), EDs are burdensome in terms of significantly impaired health-related quality of patients’ lives [8], not only because of the impact that the disorder can have on all body systems. The lifetime prevalence of any threshold ED among adolescents and young adults is 2.9% among women and 0.1% among men [9]. In 2020, there were 5167 patients with EDs treated in outpatient departments and hospital admissions, and the number has increased by approximately 15% in 10 years. Most patients (87%) were women and girls [10]. However, these statistics do not include people with EDs who seek help without a psychiatric context (eg, clients of nonprofit organizations) or, for instance, people who do not seek help at all. Both male and female patients with ED have high rates of psychiatric comorbidity [11]. These disorders are challenging for caregivers within the family system, as well as for health care professionals [12,13]. Moreover, EDs are egosyntonic—the person sees the disorder as part of themselves, might attribute positive valuations to ED consequences, and may perceive ED not only as an illness but also as a meaningful behavior [14]. Recovery from an ED is a long and potentially life-threatening process [15]. Despite the severity of the illness, patients with ED symptoms do not necessarily seek treatment [16].

At different levels of illness, patients have different motivations and goals for changing their ED-connected behavior. The stages of change model [17,18] proposes 6 stages of motivation for recovery. This model is particularly relevant to understanding the phases of EDs as relapses are regarded as integral parts of the change cycle, and an application to the ED context has been examined [18]. The first 2 stages are described as precontemplation (ie, nonexisting or limited intention to change behavior) and contemplation (ie, willingness to think about the change but not commit to it). These 2 stages resemble the earliest phases of ED as bounded by limited or lacking motivation to change ED-connected behavior. Following are the stages of preparation (ie, intention to change the behavior), action (ie, actively modifying the behavior), maintenance (ie, work on the prevention of relapse), and termination (ie, zero temptation to relapse). This study presumes that people with EDs often switch between stages of illness, although the distinction between these stages might be blurred or even overlapping. Moreover, this study acknowledges that motivation plays an important role in the behavior of people with ED experience, including web-based praxes, and that motivations differ with respect to the stage of illness.

Prior research has explored the role of the internet and communication technologies in the lives of people with ED experience from various perspectives. On a general level, media are studied as one of the sociocultural risk factors that contribute to the etiology of EDs through the cultural ideals of appearance and weight [19]. Other lines of research have focused on exposure to ED-promoting websites. Such exposure is associated with a reduction in the number of calories, restriction in food consumption, greater body dissatisfaction, and a greater drive for leanness and musculature, especially among vulnerable individuals [20]. In contrast, the treatment of EDs has also moved to the web-based environment, as the anonymous internet provides a safe environment for people with EDs who may experience high levels of secrecy, shame, and stigma [21]. Internet-based treatments may offer self-assessment, self-monitoring, information about EDs, and advice for systematic treatment plans. People with ED experience also use the internet and visit web-based groups to seek information, get support, and share experiences [22].

### Information Evaluation in the Web-Based Environment

Although tools for the assessment of web-based health information have been developed (eg, a study by Beaunoyer et al [23]), to the best of our knowledge, little attention has been paid to how web-based information is evaluated by users with health-related problems. This study aims to fill this gap by examining the information quality assessment by people with ED experience within their specific ED-relevant web-based activities. The construct of information quality is broad and lacks conceptual clarity. For instance, Tao et al [24] distinguished 5 dimensions of information quality on health websites: completeness, understandability, relevance, depth, and accuracy. However, other indicators of the quality of web-based information have also been identified, such as perceived aesthetics, credibility, reliability, security, consistency, usefulness, and worth [25-27]. This study defines information quality assessment as an evaluation of web-based information and materials, with a focus on both web content (including web-based information and features) and its design (the representation of the content for users) [28]. Accuracy, currency, and credibility may be among the content quality criteria, and aesthetics, cultural sensitivity, or accessibility be among the design-related criteria [29]. However, the definition of *quality* can also differ interindividually depending on the specific context and purpose of the information.

Another presumption of this study is that the web-based environment is highly diverse and can be assessed differently in terms of quality. For example, an environment (presumably) controlled by expert editors, such as websites, can be perceived as more credible than a personal blog [30]. User characteristics, such as personal traits, abilities, previous knowledge, and topic familiarity, are also considered. The assessment process also depends on interindividual differences between users and the perceived type and context of information [31-34]. For example, the dual processing model of credibility focuses on individual factors and depicts how factors independent of message quality can affect our evaluation [35,36]. According to this model, when the user has the motivation and ability (eg, the level of literacy skills) to evaluate the quality of information, they are likely to use an analytical strategy to assess credibility systematically and rigorously. The lack of motivation inhibits users from putting effort into credibility evaluations. However, if they lack the ability and yet have motivation, they will rely more on peripheral cues (eg, the appearance of the site) and heuristics to form a judgment. Such heuristics can take the form of relying on the reputation of the source or endorsement from others [37].

### User Characteristics Connected to EDs

Although research evidence about the specifics of web-based information evaluations made by people with ED experience is limited, their assessment may, in some moments, contrast that of people without ED experience. For example, their motivation for evaluation may differ according to the current state of their illness (ie, affected by the egosyntonic feature of the illness, where people with ED experience might see information congruent with their pro-ED values). Thus, to understand how people with EDs process and evaluate information, it is crucial to consider their psychological traits and cognitive characteristics. For example, a review by Cassin and von Ranson [38] revealed that both AN and BN are characterized by perfectionism, obsessive compulsiveness, neuroticism, negative emotionality, harm avoidance, low self-directedness, low cooperativeness, and traits associated with avoidant psychiatric disorders.

Moreover, some of the cognitive challenges may affect how people with the experience of EDs process web-based and offline information. Current research has demonstrated an attentional bias for disorder-salient stimuli (related to food and the body), which indicates that people with ED experience have a potential overall deficit in processing conflicting information [39]. Another cognitive deficit is a weak central coherence when attention is focused on detail, resulting in global understanding deficits [40]. Furthermore, poor set shifting (ie, a lower ability to move back and forth between tasks) results in cognitive inflexibility [41,42]. This inflexibility may manifest in rigid and concrete problem solving, reliance on strict habits and rules, and difficulties with multitasking [43].

### Aims of the Study

To the best of our knowledge, no study has focused on the evaluation of web-based health information in the context of ED topics among people with an ED experience. This qualitative study intends to enhance knowledge about this topic and help us understand how women with ED experience evaluate web-based information. This study uses previous knowledge related to information evaluation. Specifically, it presumes that individual characteristics, including personality, abilities, and motivation, shape the formation of judgments about information. Moreover, information assessment can be more or less thorough. Finally, this study considers that the web-based environment provides different cues that may be used in the process. On the basis of this knowledge, the following research questions were formulated: what ED-related characteristics of young women with the experience of EDs are involved in their evaluation of web-based information, and what criteria do young women with the experience of EDs use in evaluating web-based information?

## Methods

### Recruitment

The data were obtained from a research project that examined the role of new technologies among young people with ED experience in the Czech Republic. Respondents were recruited via leaflets handed out at universities in large Czech cities and in the waiting rooms of health care professionals working with people with EDs (mainly in hospitals and ED-focused nonprofit organizations in the Czech Republic). Owing to the seriousness of the illness, some respondents were available only via web-based means. Thus, the outreach was gradually expanded during the sampling process, with invitations to participate on websites relevant to EDs (both supporting ED behavior and the treatment of EDs). From the previous quantitative part of the research [44], we had a list of 307 Czech websites (including blogs and Facebook groups) that focused on healthy lifestyles (including fitness and nutrition) and professional help for EDs and promoted ED behavior (mainly pro-ED blogs and groups). These websites were found via search engines using keywords connected to a healthy lifestyle (ie, exercise, diet, and healthy eating), ED problematics (ie, professional help and informational websites), and ED promotion (ie, keywords identified in previous pro-ED research, such as *pro-ana*, *thinspo*, and *bonespo*). Finally, we posted an invitation on 15 websites (including Facebook groups) that focused mainly on ED information, recovery, and ED promotion and that ranked highest in website traffic. Research has shown that EDs are most prevalent among women [9], and risk factors are present in early adolescence, although anorexia and bulimia tend to emerge in late adolescence and early adulthood. However, the onset of EDs is individual [45]. Thus, the criterion for respondents was to be aged between 13 and 28 years and have experienced (now or in the past) a form of an ED.

### Sample

The final sample comprised 30 Czech women aged 16 to 28 (mean 22.4, SD 3.9) years. Although EDs are increasing among men [46], and we actively recruited respondents of all genders, the recruitment of men was not successful. All participants claimed to experience or have experienced various EDs (AN 13/30, 43%; BN, 3/30, 10%; binge ED 1/30, 3%; or multiple ED diagnoses 13/30, 43%). Some of them had reached out for the help of health professionals and had an official diagnosis (27/30, 90%), whereas others did not (3/30, 10%). Respondents reported the presence of an ED in their current life (22/30, 73%) or that they were in full recovery (8/30, 27%). Experiences with the illness varied from 1 to 16 (mean 6.3, SD 4.5) years.

### Procedure

A total of 30 semistructured interviews were conducted face to face (23/30, 77%) or via Skype web-based sessions (7/30, 23%), which lasted 41 to 118 (mean 61.0, SD 21.1) minutes. The interviews focused on the use of new technologies, including questions about the role of the internet in respondents’ lives; for example, “What helps you orient in health-related online information?”; “From where do you retrieve the online information?”; and “What are the most common online activities?” The interviewers also asked about the criterion for information relevance and quality and on what cues respondents adopted the information and acted on it.

All participants were informed of the ethical aspects and purpose of the research, and they provided written informed consent. In the case of respondents aged <18 years, parents provided written consent. The interviewers had psychotherapy training and at least 2 years of psychotherapy practice as a condition for reducing potential stress among respondents.

### Ethics Approval

This study was approved by the Ethical Committee of the Faculty of Social Studies of the Masaryk University, Brno, Czech Republic.

### Data Analyses

The thematic analysis developed by Braun and Clarke [47] was used as the analytic method and was conducted by the first author of the study (HD). The inductive approach of analysis was used when the themes were content (data) driven and emerged from the interaction between the researcher and respondents, regardless of the specific questions. Therefore, researchers could capture the complexities of meaning within a text and understand the more tacit content.

During the analysis, we were inspired by the steps in the guidelines presented by Braun and Clarke [47] and Guest et al [48]. First, researchers became familiar with the data and text segmentation by rereading the transcripts and noting their initial ideas in a logbook. Passages related to information evaluation were segmented. In creating the initial codes, the authors entered the transcripts into the qualitative analytic software NVivo (version 10) and started to generate a codebook for codes and their labels. The code represents the specific, interesting, and essential elements of the text, and it has a greater level of abstraction than the themes [47,48]. The labels of these codes were mainly in the form of in vivo phrases used by respondents and a simplified description of the code content. These codes were discussed during research team meetings to create common categories for an initial category structure. Subsequently, continuing the analysis of the second half of the transcripts, the authors merged the codes into subthemes based on their similarities. Next, revisions were made to avoid the overlapping of the meanings. According to Chang et al [49], researchers included only subthemes with ≥3 respondents to prevent fragmentation. During the phase of defining and naming the themes, the authors reread the existing codes to better understand their meaning and created corresponding labels and descriptions. Saturation was reached, with the occurrence of redundancy, after 30 interviews. The last 4 interviews confirmed saturation of the themes as they did not create new categories. Subsequently, the researchers checked whether the themes and subthemes were internally coherent and consistent. As some of the subthemes still overlapped, they merged some such subthemes. In addition, the themes were renamed to better correspond to their meanings. The final list of themes and subthemes is presented in the *Results* section.

The following steps were applied to ensure the validity of the results. First, all researchers followed an interview guide to standardize the data collection. Furthermore, remarks regarding the content of the interviews were discussed. Second, the first (HD) and third author (MS) consulted on emerging themes during the entire analytical process. Third, the study’s third author (MS) conducted an audit comprising reading the related parts of the data and validating the final analyses. Fourth, the second author (HM) advised on the final presentation of the results to clarify the meaning of the themes. Examples supported the transparency of the interview results. Finally, the researchers applied the verbal labels of frequencies in the *Results* section to state how many respondents mentioned a particular subtheme. Instead of using the number of respondents, a verbal label (a pronoun connoting an indeterminate quantity) was attached to enhance the qualitative methodology. Inspired by the verbal counting of Sandelowski [50], the researchers operationally define, for example, *few* as something occurring among 3 to 8 respondents (see the *Results* section).

## Results

### Overview

A total of 4 themes and 10 subthemes were identified, as summarized in Table 1. The theme *respondent characteristics* represents respondents’ characteristics that influenced their information evaluation. Themes of *content cues*, *characteristics of other users*, and *website cues* present the respondents’ cues mentioned in their evaluation. The results cover the findings relevant to the entire information assessment process. It encompasses the initial phases of information seeking, including factors that affect preferences for diverse sources and further evaluation of the found information.

The specifics of the EDs phases emerged as important aspects of respondents’ characteristics. Some respondents spontaneously categorized themselves as being in the *disorder stage* or in the *treatment process* during the interviews. Respondents who described themselves as being in the *disorder stage* had statements and descriptions that fit in the precontemplation and contemplation stages of the stages of change model [17], whereas those seeing themselves as being in the *treatment process* described more processes connected to the preparation, action, maintenance, and termination stages of the model. These dimensions were emphasized for each theme and subtheme, as shown in Table 1.

**Table 1:** The final list of themes and subthemes and their occurrence in ED^a^ phase^b^.

Theme and subtheme		ED phase (disorder stage and treatment process)	Frequency label
**Respondent characteristics**			
	Motivation	Both	Most
	Abilities and resources	Both	Few
	Congruence between personal experience and information	Both	Some
**Content cues**			
	Verification	Both	Few
	Novelty	Only in the disorder stage	Few
	Social information pooling	More in the disorder stage	Some
**Characteristics of other users**			
	Source expertise	Both	Most
	Similarity to respondent	Both	Most
**Website cues**			
	Reputable look	Unclear	Few
	Photographs of people relevant to ED	More in the disorder stage	Some

^a^ED: eating disorder.

^b^Frequency labels in Table 1 and further in the text describe how many respondents mentioned each subtheme. *Few* indicates 3 to 8 respondents, *some* indicates 9 to 17 respondents, *most* indicates 18 to 29 respondents, and *all* indicates 30 respondents.

### Respondent Characteristics

#### Overview

This theme captures how the characteristics of respondents, including their web-based behavior patterns, are involved in information evaluation. Specifically, it addresses how respondents arrived at certain web-based information and how they evaluated it. The following subthemes emerged: *motivation, abilities and resources,* and *congruence between personal experience and information.*

#### Motivation

Most respondents described 2 main motivations relevant to their illness: maintaining their disorder in the disorder stage or getting cured during the treatment process. The particular type of motivation affected what information the respondent chose, as one respondent revealed the following:

If someone, I think, has the motivation, that she can cure herself, then she is able to filter on the internet to what she wants to read, what she doesn’t want to read, and what she lets influence her and what not.

R13

For respondents in the disorder stage, the vision of a skinny body was so strong that they looked up and accumulated as much information as possible to maintain their goals. However, they were not concerned with its evaluation. They read and eventually “tried everything” (R2). One of the respondents piled up information instead of analyzing it, and although the information was labeled as nonsense, they read and used it anyway. They also talked about their passive role in choosing information when their disorder decided what was needed. A respondent experienced the following:

I know what is right, what is really right, I know it. But of course, anorexia chooses what she likes, not how it’s supposed to be like. So I believe more or less in almost everything.

R29

In contrast, respondents in the treatment process did not read or seek proana and promia information as they were afraid of being pulled back into the disorder stage. However, they described their persistent sensitivity to disordered relevant information, such as diet commercials. Nevertheless, they used different information checks, as a respondent pointed out the following:

When the person is in an acute stage, then she blindly follows what she wants to gather. Now I follow what I want to gather, that I want to be healthy. But in that acute phase, I don't think about if it will hurt me. And now I want to verify all information with someone responsible, with a professional who tells me “it is appropriate for you, it is not appropriate for you.”

R6

#### Abilities and Resources

For a few respondents, their current situation in terms of abilities and resources affected their information selection. For instance, respondents at the disorder stage chose exercise and weight reduction tips based on their physical state*.* Financials played a role as well, leading to choosing diet tips and menus suited to their monetary situation. One of the respondents in the disorder stage also mentioned that the criterion for information selection was the time spent applying particular advice to her life.

Information choice was also influenced by the information-seeking strategies and skills of the respondents. Some actively used search engines and keywords, such as *anorexia*, *bulimia*, and *eating disorders* or questions such as “How to do away with bulimia” (R2) or “How to throw up” (R4). They then mainly clicked on the first link. Active searches also included clicking on links on blogs and forums that led to similar websites.

For others, disorder-related information had appeared unwelcomely when information was “jumping out” (R10) at them against their will. Respondents discussed how the information was “attacking them” (R27) and how it was almost impossible to avoid. For respondents in the treatment process, it was proana or promia blogs or information about eating. For respondents in the disorder stage, it was information about treatment or dieting.

#### Congruence Between Personal Experience and Information

The knowledge and experience gathered through their disorder helped some to assess the relevance of information about EDs. The information that corresponded to personal experiences was credible and relevant. For example, respondents in the disorder stage said that they did not trust diets as they already knew what weight loss strategies were good for them:

I was always using my experience. So, for example, when I knew that by that [caloric] intake I had lost weight, or by eating that food I had lost weight, then I simply ate it, because I had tested it. And I did not trust anything else.

R8

Respondents in the treatment process viewed healthy lifestyle information as appropriate because of their experience with professionals and with new nutrition information.

### Content Cues

#### Overview

According to the respondents’ evaluation of information, content cues described the qualities (in the sense of features) of the information found on websites. The following subthemes emerged within this theme: *verification*, *novelty*, and *social information pooling*.

#### Verification

Information was approved by a few respondents when it was consistent across websites.

However, some respondents needed to verify the web-based information in the offline environment by comparing the information with books or, as mentioned by respondents in the treatment process, via consultation with professionals.

#### Novelty

Another cue for some respondents was whether the information was new. This subtheme was mentioned more by respondents in the disorder stage:

How should I behave towards the food, how to hide the food, what should I avoid, how to deal with various situations, what to do and what not to do. Any new information was good information for me.

R27

Consequently, other users’ long-term sharing of new posts was considered beneficial rather than the sharing of a few posts once in a while: information posted “every day or every other day was more credible” (R25).

#### Social Information Pooling

The subtheme most cited by some respondents was how others’ experiences helped them judge information. This subtheme was mentioned more by respondents in the disorder stage and represented sharing opinions, recommendations, and feedback on desirable topics between respondents and other users (eg, comments below articles, reactions on forums, and liking some posts via social network sites). Respondents saw others’ recommendations as helpful and worthy, although they had never met them on the web or offline:

It is weird that I took their advice a lot. One doesn’t know the other person at all, but still follows what is written there.

R7

However, other respondents saw recommendations as valuable if they knew the users from the web-based environment (eg, following the blog of a friend).

In this process, the number of reactions was also significant. Testimonials, positive responses, and the number of *thumbs-ups* increased the chance of seeing information as trustful, whereas their absence had the opposite effect:

I read the comments if [pro-ana advice] works or not. But if there was no comment, then I did not trust it. Or I wouldn’t try it unless there was something written there, some opinion.

R7

For respondents in the disorder stage, if some information worked for others (ie, was used and acted upon), it was good:

But when she reads “try this and that,”’ then she says to herself “when others do it, it must be really cool.”

R4

For respondents in the treatment process, social pooling was essential for the assessment of the treatment procedures and practice of helping professionals.

### Characteristics of Other Web-Based Users

#### Overview

This theme captures how other users’ attitudes, experiences, activities, and personal features are involved in the information evaluation of these users and the information presented by them. Other users were mostly seen as post contributors or members of web-based communities. Their characteristics were expressed in the subthemes of *source expertise* and *similarity to the respondent*.

#### Source Expertise

The perception of who is an expert in an ED field served as a hint for information assessment. Respondents distinguished whether the information was provided by users who are currently experiencing or had experienced EDs or by ED specialists. The specialists were mentioned without further explanation or specified as professionals, such as psychologists, physicians, and nutrition specialists, and were connected to institutions, such as universities, hospitals, and ED treatment centers.

The most mentioned aspect for most respondents was whether users had experience with EDs. Those who did were seen as experts in ED problems and credible as they better understood the respondents’ issues and feelings. Respondents who preferred this expertise sometimes set experience with EDs as a cornerstone:

Certainly, there were some important basic factors. That person had to have an eating disorder, or at least not be OK with food, such as people with obesity.

R24

Specifically, one of the respondents in the disorder stage said that she ignored notes by professionals on a self-help website and read only the text passages written by people with EDs.

In both stages, the mentoring and labeling of respondents by professionals discouraged respondents from looking up and reading the information presented by these professionals. Professionals could not be trustworthy for a respondent in the disorder stage as they wanted her to gain weight and, therefore, did not provide complete information*.* In contrast, the entire community of users with ED experience was considered trustworthy. The goodwill of this community was described as being open, welcoming, and accepting, creating the feeling of an alliance.

The valuable characteristics of ED specialists on the internet were described simply as being professional, providing verified information to respondents, understanding EDs, having healthy opinions about EDs, and being selflessly helping respondents. Those who saw professionals as credible (although some questioned their expertise) perceived the ED information written by experts and treatment groups supervised by them as trustworthy.

For some respondents, the view of the expertise cue had changed over time. Those who started treatment looked more or only for information written and shared by professionals. Respondents uncertain about their willingness to be cured could have ambivalent feelings, as one respondent pointed out the following:

If I listened to the doctor, I would eat well. I know she gives me the right advice, that she is a professional. However, unfortunately, I adopt what suits me more. Rather from the Internet.

R29

#### Similarity to Respondent

Most respondents wanted to know many details about other users to better evaluate whether they were similar and, consequently, whether they were trustworthy, which in some respondents led to trying to find as many details as possible.

Respondents specifically mentioned similarity cues connected to their illness, including the same type of ED, a similar stage of the disorder, similar problems, and similar attitudes toward food. The cues not strictly linked to ED were described as similarities in current mood, humor, age, and writing style.

### Website Cues

#### Overview

This theme reveals a representation of the visual look and graphic components of the website. Two subthemes were identified: *reputable look* and *photographs of people relevant to ED*.

#### Reputable Look

Respondents named a reputable look as a good sign for further selection. This look was not defined by the specific features of a website but rather by broad general characteristics, such as clarity, lack of bias, and good organization.

#### Photographs of People Relevant to ED

Pictures, videos, advertisement photos of diet products, and especially photographs of people with experience of EDs were important for respondents, mostly in the disorder stage. Before and after images helped respondents assess the actual effect of others’ aims and provided tips to lose or gain weight. Moreover, the photographs acted as proof that the people in them were real and not lying. Specifically, respondents in the disorder stage believed in what was presented in the photographs:

I saw a picture of a woman with a gorgeous figure, so I believed that she is on that diet [from a commercial].

R23

A few respondents mentioned that they no longer took advertisement information for granted when they shifted to the treatment stage of ED.

## Discussion

### Principal Findings

The purpose of this study was to explore how young women with ED evaluate web-based information and how the specifics of their illness contribute to this evaluative process. Respondents mentioned several cues for information assessment within the websites’ content, the characteristics of other users, and the website characteristics while stressing the influence of the current phase of their illness.

First, respondents’ characteristics played an integral role in the evaluation, intervening in the entire process from exposure to information to final judgment. As Hargittai et al [32] suggested, obtaining information and its evaluation are more often handled as 2 separate research interests. However, our respondents holistically depicted these 2 steps. They explained how their *abilities* and *resources* affected this process and, importantly, what role *motivation* played in grounding their illness. *Motivation* turned out to be a vital part of the assessment, confirming the justified emphasis within the dual processing model of credibility by Metzger [36] and the stage of change model [17]. For respondents in the disorder stage, the motivation to lose weight was their biggest goal. They were not concerned about information evaluation and saw every piece of information as automatically good. This strategy could be a consequence of their *congruence between personal experience and information* (ie, EDs): the more experienced the respondents were, the more certain they were about the accuracy of their information selection. Another explanation for the automatic assessment of information may be cognitive rigidity, which is more pronounced in people with ED experience (eg, see the study by Tchanturia and Hambrook [43]). As a result, when a particular type of information is already assessed as quality, additional information from a similar topic can also be considered appropriate without further evaluation. Although such quick evaluation strategies are convenient, they may generate bias. A study by Guardiola-Wanden-Berghe et al [51] suggested that the information quality of websites on dieting and EDs was poor. Viewing them as automatically believable may lead to the risk of adopting and behaving on harmful information.

The social element attached to quality cues was also prevalent throughout most themes. The *characteristics of others* was the most frequently mentioned theme, although web-based sources and other users are often masked or missing for evaluation [35]. However, reliance on social features is part of the evaluative process, as shown in other studies [37]. Similarly, *social information pooling* was a social-connected cue whereby other users’ opinions and feedback were hints for information selection and evaluation. In particular, for respondents in the disorder stage, the information confirmed and approved by other users was seen as trustworthy. Weight reduction was the primary goal for respondents in the ED stage; therefore, they looked for fast and effective weight reduction information. However, this search could be limited in 2 aspects: as the subtheme *abilities and resources* revealed, they were tired and probably less inclined to put much energy into it; in addition, some of them did not perceive health professionals as credible. Thus, the opinions and reviews of others served as quick and less effortful tools for information evaluation. The more those testimonials were presented, the more chance the information was viewed as valid. These assumptions are similar to the bandwagon heuristic of Sundar [52], whereby people suppose that if many others think something is correct, then it must be. However, heuristics may lead to problems with crowd behavior, especially for young people with EDs, who are at greater risk of peer pressure [53]. Moreover, quality may be falsely connected to popularity when unpopular topics and information are discarded [2]. In addition, popular users may be perceived as falsely trustworthy. Thus, when and from whom people with EDs seek support and advice might be crucial for understanding their information evaluation and selection.

*User ED expertise* was another socially relevant cue in which information from others who had experience with EDs was perceived as appropriate. Boero and Pascoe [54] suggested that other users in pro-ED communities use their experiences to demonstrate their authenticity and offer advice about, for example, how to get through recovery programs without actual recovery. They also answered the questions of new members using their nutritional and medical knowledge. The respondents’ positive perception of the information presented by these users may be partly understood by the Situated Identity Enactment Model of Cruwys et al [55], highlighting the role of socially bonded norms, identity, and context. Being a member of a social group may moderate conformity with the group’s social norms. For example, these norms may be to follow a lifestyle presented by the group or to trust more experienced members. Being a member of an ED-experienced group is also vital in the treatment stage because of the transformation of illness identity to treatment identity [56]. If a member fails to follow the norms, they may be rejected [57]. Thus, one of the possible group norms may be to see ED-experienced group members as reliable, and respondents comforted by this norm seek to avoid group rejection. Studies suggest that communities of ED-experienced users represent a safe place for sharing experiences and a means of support and understanding [58]. The respondents in this study indeed saw their ED-related web-based communities as full of goodwill and, thus, were trustworthy.

The similar stage of an ED or the same ED type was another marker for user quality, reflecting studies showing that people seek and see information that matches what they already know [59,60]. Accordingly, respondents stated that users who were similar to respondents and supported respondents’ opinions might have similar opinions and are plausible. Metzger and Flanagin [2] described a self-confirmation heuristic whereby credible information confirms pre-existing beliefs. In the case of an ED, this heuristic may enhance the egosyntonic feature of the illness, whereby respondents value their ED and see the information as good if it supports its maintenance. This confirmation of established opinions may place a burden on treatment, especially at the disorder stage. In the disorder stage, respondents ignored health professional advice and followed prodisorder tips, which enabled them to pursue an ED lifestyle. In contrast, similar experiences of recovery and treatment strategies may be supportive for those who decide to treat themselves. Consequently, health professionals should be aware of the identification needs of their clients and how their selectiveness may influence what is considered credible.

Finally, respondents also mentioned cues related to websites (specifically the reputable look and photographs associated with EDs); however, mentions of such elements were relatively scarce. This could suggest that, within the self-report context of the testimonies, respondents consciously reflected that website cues were not as significant as to be named and instead focused on social aspects in the overall evaluation.

### Limitations of the Study

This study had several limitations. First, the sample was relatively homogenous as it comprised only women, although they differed in age, region, and illness experience.

Thus, the testimonies might not represent other demographics, such as men or different ethnicities. Next, the respondents reported the condition of the ED diagnosis, and not all of them had experience with professional health care. It is possible that although respondents claimed to have an ED themselves, they would not meet the diagnostic criteria of psychological diagnostic manuals, such as the Diagnostic and Statistical Manual of Mental Disorders. Similarly, experience with technology was not measured and might have varied for different illnesses and ages. In addition, the 2 stages of EDs (disorder vs treatment process) derived from the respondents were often blurred, unclear, sometimes overlapping, or even missing. Although some processes of these stages might resemble the stage of change model [17], respondents were not further asked for their motivation to change. Finally, respondents’ browsing history was self-reported, and researchers did not retrieve the browsing history data. Thus, respondents’ internet use and information were unique to each and based and customized on their previous web-based activities, which can vary substantially among individuals. For future studies, we suggest using more accurate measures to use the level of change and consider different contexts and settings for respondents’ information evaluation.

### Conclusions and Implications

This study showed that the web-based information evaluation reported by women with the experience of EDs is a complex process. The assessment is influenced by current personal characteristics related to the illness (mainly the motivation to maintain or cure the ED) using cues associated with information content, other users, and website look. The study findings have important implications for health professionals, who should ask their clients questions about web-based communities and their needs to understand what information and sources they choose. They should support clients by consulting their judgments and uncertainties about information evaluation. Further investigation into the role of consulting web-based information with professionals in information assessment by users might benefit future therapeutic practices. Who and what is positive regarding quality and how it changes within the ED phases may help to understand the illness. Moreover, quality cues may serve as merits for designing an ideal website for health providers who use the internet for prevention and intervention. Future research might benefit from the experimental design of these websites and their evaluation by people with ED experience. For example, the personal stories of people experiencing EDs, their tips about treatment, and web-based peer groups on websites may increase the perceived quality of information and providers. However, the perceived quality of information might not be associated with the direct application and use of this information in an offline environment. Thus, the dynamism of the transfer between perceptions of information quality and acting on this information might be another research interest for the future.

## Abbreviations

AN: anorexia nervosa
BN: bulimia nervosa
ED: eating disorder
